# RNAi downregulation of three key lignin genes in sugarcane improves glucose release without reduction in sugar production

**DOI:** 10.1186/s13068-016-0683-y

**Published:** 2016-12-20

**Authors:** William P. Bewg, Charleson Poovaiah, Wu Lan, John Ralph, Heather D. Coleman

**Affiliations:** 1 Queensland University of Technology, Brisbane, QLD 4000 Australia; 2 Department of Biology, Syracuse University, Syracuse, NY 13244 USA; 3 Department of Biological Systems Engineering, University of Wisconsin, Madison, WI USA; 4 US Department of Energy, Great Lakes Bioenergy Research Center (GLBRC), Wisconsin Energy Institute, University of Wisconsin, Madison, WI 53726 USA; 5 Department of Biochemistry, University of Wisconsin, Madison, WI 53726 USA

**Keywords:** Lignin biosynthesis, Ferulate 5-hydroxylase, Caffeic acid *O*-methyltransferase, Caffeoyl-CoA *O*-methyltransferase, Sugarcane, RNAi

## Abstract

**Background:**

Sugarcane is a subtropical crop that produces large amounts of biomass annually. It is a key agricultural crop in many countries for the production of sugar and other products. Residual bagasse following sucrose extraction is currently underutilized and it has potential as a carbohydrate source for the production of biofuels. As with all lignocellulosic crops, lignin acts as a barrier to accessing the polysaccharides, and as such, is the focus of transgenic efforts. In this study, we used RNAi to individually reduce the expression of three key genes in the lignin biosynthetic pathway in sugarcane. These genes, caffeoyl-CoA *O*-methyltransferase (*CCoAOMT*), ferulate 5-hydroxylase (*F5H*) and caffeic acid *O*-methyltransferase (*COMT*), impact lignin content and/or composition.

**Results:**

For each RNAi construct, we selected three events for further analysis based on qRT-PCR results. For the *CCoAOMT* lines, there were no lines with a reduction in lignin content and only one line showed improved glucose release. For *F5H*, no lines had reduced lignin, but one line had a significant increase in glucose release. For *COMT*, one line had reduced lignin content, and this line and another released higher levels of glucose during enzymatic hydrolysis. Two of the lines with improved glucose release (F5H-2 and COMT-2) also had reduced S:G ratios.

**Conclusions:**

Along with improvements in bagasse quality for the production of lignocellulosic-based fuels, there was only one line with reduction in juice sucrose extraction, and three lines with significantly improved sucrose production, providing evidence that the alteration of sugarcane for improved lignocellulosic ethanol production can be achieved without negatively impacting sugar production and perhaps even enhancing it.

**Electronic supplementary material:**

The online version of this article (doi:10.1186/s13068-016-0683-y) contains supplementary material, which is available to authorized users.

## Background

Sugarcane is a key global agricultural crop with high production rates. After the extraction of the juice for which it is grown, the remaining plant material is often used inefficiently for the production of energy through burning. However, this lignocellulosic material could be used for the production of biofuels, adding value to an existing commodity [1]. Challenges remain in the cost-effective production of cellulosic ethanol and this is due in large part to plant cell wall recalcitrance. In particular, lignin can pose challenges for accessibility of polysaccharidase enzymes to the cell wall polysaccharides, and as a result, it has become a key focus of biotechnology efforts. There have been successes in other species demonstrating that the downregulation of genes within the lignin biosynthetic pathway is an effective way of reducing lignin content and/or altering structure in ways that improve the digestibility of plants for biofuel production [2–8].

There are many genes involved in the production of lignin monomers from phenylalanine [8–10]. Caffeoyl-CoA *O*-methyltransferase (*CCoAOMT*) is one of the key enzymes in the synthesis of *G* and *S* monomers of lignin [11]. Previous work has shown that the downregulation of *CCoAOMT* results in decreases in *G* monomers in alfalfa [12–14], pine [15], maize [16], flax [17], poplar [18, 19], and tobacco [20]. Reduced *CCoAOMT* expression has also been shown to result in increased efficiency of enzymatic hydrolysis in alfalfa [12] and Arabidopsis [21]. In softwoods, but not in hardwoods or other dicots, *CCoAOMT* downregulation results in the incorporation of caffeyl alcohol, as a lignin monomer, into lignins and resulting in benzodioxane structures in the polymer [15].

A second gene, ferulate 5-hydroxylase (*F5H*) encodes for an enzyme that performs a necessary step in the production of the S lignin monomer [22]. A decrease or increase in F5H expression has been found to reduce or increase, respectively, the S monomer presence accordingly in alfalfa [13, 23, 24], poplar [25], and Arabidopsis [21, 26].

A third key gene in lignin biosynthesis is caffeic acid *O*-methyltransferase (*COMT*), which also plays an important role in monomer composition [22, 27]. RNAi downregulation of *COMT* expression in canola [28], alfalfa [12, 13], switchgrass [4, 29, 30], and sugarcane [31, 32] consistently resulted in decreases in *S* monomers, as well as an overall decrease in total lignin content. Similar results have been found after sense or antisense downregulation of COMT in alfalfa [14, 23, 33, 34], poplar [35], tobacco [20], and maize [36, 37]. More importantly, COMT-deficiency results in the incorporation of a novel monomer, 5-hydroxyconiferyl alcohol, derived from the precursor to COMT, 5-hydroxyconiferaldehyde, via CAD reduction; the resultant 5-hydroxyguaiacyl benzodioxane units can represent a substantial proportion of the lignin polymer [38, 39]. Downregulation of COMT expression was linked to improved enzymatic hydrolysis in alfalfa [12], Arabidopsis [21], switchgrass [4, 29], and sugarcane [31, 32].

Here we focus on the RNAi downregulation of three lignin biosynthetic genes: *CCoAOMT*, *F5H* and *COMT*. These genes were selected for their potential roles in directing metabolites to specific lignin monomers, as well as for their location in the phenylpropanoid pathway, which may help reduce the chance of detrimental phenotypes that can occur when genes early in the pathway are downregulated [40–43].

## Methods

### Generation of RNAi constructs

The sequence for *COMT* was available (AY365419; AJ231133), and *CCoAOMT* and *F5H* were generated from available sugarcane EST databases (*CCoAOMT*: CA168805, CA071322, CA159865, CA180815, CA159865, CF575000, CA279207, CA179873; *F5H*: CA185931, CA134666, CA135938, CA287472, CA278023, CA253395, CA103877). Gene segments of approximately 400 bp in length were amplified from sugarcane cultivar KQ228 for each of the three genes of interest (Additional file 1). These amplicons were used to generate RNAi hairpin constructs with a synthetic intron that were ligated into an existing entry vector between a maize ubiquitin (Ubi) promoter and intron (iUbi) [44, 45] and the nopaline synthase (nos) terminator [46]. The three *Zm*Ubi-iUbi-sense/syntron/antisense-nos/pBS RNAi vectors were confirmed by sequencing.

### Generation of transgenic sugarcane

KQ228 callus was co-bombarded with individual RNAi/pBS constructs and *Zm*Ubi-iUbi-nptII-nos/pUC19. Transformation control callus was bombarded with *Zm*Ubi-nptII-nos/pUC19 only and hereon the regenerated plants are referred to as controls. After plant regeneration from callus as previously described [47], individual events were transferred to growth chambers for continued development. They were maintained under 16 h days, at 25 °C and were watered every 2 days.

### qRT-PCR analysis

For initial qRT-PCR screening, RNA was extracted from leaf tissue of 3-week-old growth chamber-acclimatized sugarcane plants. Transgenic plants representing a spectrum of expression levels of the RNAi targeted gene and control plants were transferred to the greenhouse once they were approximately 30 cm in height. Plants were grown at 27 ± 3 °C in 4.5 L pots under natural light. They were watered to saturation twice per week and fertilized once per month with regular tiller removal. Following greenhouse growth, RNA was extracted from young and maturing stem internodes for qRT-PCR analysis. Plants for further analysis were selected based on the lowest expression levels of the targeted lignin biosynthesis gene in mature internodes.

RNA was extracted using Tri Reagent^®^ (Sigma-Aldrich) following the manufacturer’s protocol. Extracted RNA (1 µg) was digested with RQ1 RNase-free DNase (Promega) following manufacturer’s methods with a 37 °C incubation of 1 h. DNase-treated RNA was used as a template for first strand cDNA synthesis in the M-MLV Reverse Transcription system (Promega) following manufacturer’s instructions and using an oligo-dT primer. Samples were analyzed using a Qiagen Rotor-Gene Q (Qiagen, Limburg, NLD), and relative transcript levels were quantified using delta critical threshold values (ΔCt) as follows: ΔCt = 2^−(Ct gene of interest−Ct housekeeping gene)^ [48]. The qRT-PCR primers (Additional file 1) were designed to amplify regions of the gene not included in the RNAi targeted sequence to avoid any amplification of the expressed RNAi construct sequence.

### Harvesting of sugarcane and phenotypic characterization

Transgenic and control sugarcane plants were grown in the greenhouse for nine months. All plants were watered to saturation two days before being destructively harvested. Harvesting occurred between 10 am and 5 pm over three consecutive days. Before measuring and cutting, leaf tissue and sheaths were removed, and the internodes were counted as described previously [49]. The length of the stalk was measured (internode 1 to the final internode); and the number of internodes and the diameter of the third internode from the base were recorded. Average internode length was calculated by dividing height by total number of internodes. For all analyses, only internode tissue was used.

### Cell wall composition

Four plants per RNAi construct with the lowest level of expression of the targeted lignin gene in maturing internode tissue were selected for compositional analysis. Tissue was prepared as per [50]. Oven-dried samples were ground to pass through a 2 mm screen and underwent successive overnight washes with water and ethanol to remove extractives [51]. Lignin and structural carbohydrates were quantified by a modified acid hydrolysis method [52]. Ground and extracted tissue was reacted in 72% sulfuric acid for 1 h at 30 °C inside a pressure tube before being diluted and autoclaved for 1 h. Acid-soluble lignin was determined by UV–Vis spectrophotometry and acid-insoluble lignin was measured gravimetrically [52]. Cell wall carbohydrates were analyzed using high-performance liquid chromatography (HPLC). A Waters (Milford, MA, USA) e2695 Separations Module equipped with a Showa Denko (Bavaria, DE) Shodex SP-0810 sugar column (85 °C) with micro-guard de-ashing columns (BioRad, Hercules, CA, USA) and a Waters (MA, USA) 2414 Refractive Index Detector was employed.

### Enzymatic hydrolysis

Three plants per RNAi construct that showed the largest difference in lignin composition relative to controls were pretreated before undergoing enzymatic hydrolysis for 72 h with six sampling time points. The RNAi lines were compared to transgenic controls that were assessed concurrently. Bagasse was ground to a fine powder before undergoing a mild pretreatment of 1% (w/w) sulfuric acid at a ratio of 10:1 with bagasse followed by autoclaving (130 °C for 30 min). Samples were then washed with water (3 × 50 mL). Enzymatic hydrolysis of transgenic and control bagasse was performed using Accellerase 1500 (Genencor). Before use the filter paper units (FPU) and protein concentrations were determined [53] to be 46.8 FPU/mL and 22.87 mg/mL, respectively.

Enzymatic hydrolysis was performed in 200 µL tubes following published methods [54]. Ground bagasse samples were mixed with 50 mM sodium acetate + 0.02% (w/v) sodium azide to a concentration of 1.3% cellulose (w/v) and rotated overnight at 4 °C. A 2× enzyme master mix was prepared containing Accellerase 1500 and *Aspergillus niger* β-glucosidase (Megazyme) to ensure complete hydrolysis of cellobiose to glucose. The final reaction concentration of Accellerase 1500 was 6 FPU (2.93 µg/g cellulose) and β-glucosidase was 50 µg/g cellulose. A low FPU in combination with the mild pretreatment was considered the best approach to highlight any enzymatic performance differences, including subtle differences, due to structural changes in the cell walls of transgenic plants compared to controls [21].

Pretreated and non-treated control bagasse samples were digested in triplicate at 50 °C with rotation for 72 h with samples being taken at 0, 6, 12, 24, 48 and 72 h. Reactions were quenched in liquid nitrogen and stored at −80 °C. The glucose released in each sample was analyzed using a d-Glucose Assay (GOPOD Format; Megazyme) following manufacturer’s instructions.

### Juice extraction and soluble sugar quantification

Quantification of juice sugar components was performed on the plants selected for enzymatic hydrolysis. Juice was hot-water-extracted from internodes ground under liquid nitrogen as previously described [55]. Samples were diluted according to ICUMSA method GS7/8/4-24 using lactose as an internal standard and quantified using high-performance ion chromatography (HPIC).

### Determination of cellulose crystallinity index in bagasse

For lines where tissue was available (one control, two CCoAOMT, and one each F5H and COMT plants), bagasse that had been finely ground in a McCrone (IL, USA) micronising mill was used to determine cellulose crystallinity index [56]. X-ray diffraction patterns were recorded at room temperature with a Bruker (WI, USA) AXS D8 Advance X-ray diffractometer from 10° to 40° using Cu/Kα_1_ irradiation (1.54 Å) at 40 kV and 40 mA. A 15 s/step scan speed with a step size of 0.05 s was used. The crystallinity index (CI) was obtained from the relationship between the intensity of the 002 peak for cellulose I (I_002_) and the minimum dip (I_am_) between the 002 and the 101 peaks using the equation: CI (%) = [(I_002_ − I_am_)/I_002_] × 100 where I_002_ = intensity at 22.7 Å and I_am_ = 18 Å. The divergence slit and anti-scatter slit were 3.722°. The program XRD commander (Bruker, WI, USA) was used to record and analyze the data.

### Preparation of samples for NMR analysis

The dried cell wall sample was pre-ground for 30 s in a Retsch MM400 mixer mill at 30 Hz, using zirconium dioxide (ZrO_2_) vessels (10 mL) containing two ZrO_2_ ball bearings (10 mm in diameter). The cell walls were extracted with distilled water (ultrasonication, 1 h, three times) and 80% ethanol (ultrasonication, 1 h, three times). The cell walls were dried and finely milled using a Fritsch planetary micro mill PULVERISETTE 7 (Idar-Oberstein, Germany) at 600 rpm with ZrO_2_ vessels (20 mL) containing with 10 ZrO_2_ ball bearings (10 mm in diameter). Each sample (200 mg) was ground for total 2 h 40 min (interval: 10 min, break: 5 min, repeated 11×). The cell walls were suspended in sodium acetate buffer (45 mL, pH 5.0), inoculated with Cellulysin™ (60 mg, Calbiochem, USA) and incubated at 35 °C for 72 h. The solids were pelleted by centrifugation (20 min, 8000 rpm). The pelleted material was collected and treated with Cellulysin a second time. After the second cellulose treatment, the pelleted material was washed three times with RO water (45 mL, ultrasonication 10 min, pelleted by centrifugation). After lyophilization, the obtained enzymatic lignin (EL, 30 mg) was dissolved in 0.8 mL DMSO-d_6_/pyridine-d_5_ (4:1, v/v) and subjected to NMR characterization.

### NMR analysis of lignin monomer composition and structure

HSQC NMR spectra of ELs in DMSO-d_6_ were recorded at 25 °C on a Bruker Biospin (Billerica, MA) AVANCE 700 MHz spectrometer fitted with a cryogenically cooled 5 mm quadruple-resonance ^1^H/^31^P/^13^C/^15^N QCI gradient probe with inverse geometry (proton coil closest to the sample). Bruker’s Topspin 3.5 (Mac) software was used to process spectra. The central solvent peak was used as internal reference (δ_C_/δ_H_: DMSO-d_6_, 39.5/2.95).

### Statistical analysis

Statistical analysis involved a one-way ANOVA with LSD post hoc analysis, *p* = 0.05 comparing transgenic plants to controls. As phenotypic measurements could only be made once per transgenic plant, the number of standard deviations (*z* scores) for each RNAi plant measurement were calculated against control plants. Measurements were considered different to controls if a *z* score greater than 2 or less than −2 was calculated.

## Results

**Fig. 1:**
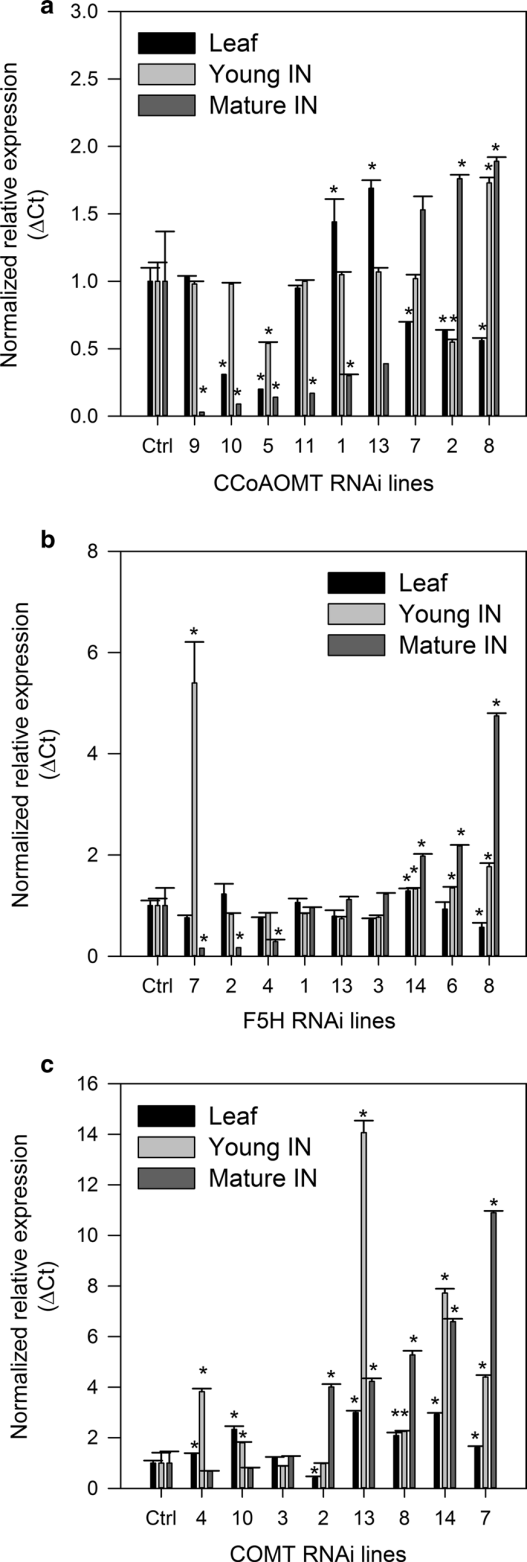
Quantitative expression of RNAi targeted genes in transgenic sugarcane. Quantitative PCR ΔCt values showing standard error of the mean of **a** CCoAOMT, **b** F5H, and **c** COMT expression in the transformed sugarcane plants after qRT-PCR analysis of leaf tissue prior to greenhouse growth, and young and maturing internode tissues post-harvest. Each sample underwent qRT-PCR in triplicate. Plants are listed in ascending total lignin content for each line. Control *n* = 3

Plants individually harboring each of the three RNAi constructs were successfully regenerated along with transgenic controls. Following nine months of growth in the greenhouse (to maximum height possible and prior to senescence), qRT-PCR was performed on young and maturing internode tissue to supplement initial qRT-PCR screens on leaf tissue carried out prior to moving the plants to the greenhouse (Fig. 1; Additional file 2). There was great variability in gene expression levels amongst events. *CCoAOMT* and *F5H* RNAi plants both showed the greatest reduction of targeted gene expression in maturing tissue with little to no downregulation observed in leaf or young internodes. *COMT* RNAi plants showed little downregulation of *COMT* expression in leaf, young or maturing tissues. In maturing tissue, only two *COMT* RNAi plants (COMT-4 and COMT-10) showed a reduction in *COMT* expression of 32 and 21%, respectively, and this was not statistically significant. The remaining *COMT* RNAi plants had expression levels higher than controls including COMT-2 with a fourfold increase in *COMT* expression in maturing tissue (Fig. 1). In contrast, the *CCoAOMT* RNAi plants had 83–97% reduction in expression and the *F5H* RNAi plants had varying levels of *F5H* reduction, from 4 to 84% (Fig. 1). Based on the gene expression data in maturing internodes, we selected four plants per line for further analysis.

### Phenotypic measurements

Phenotypic measurements were taken at the time of harvest. The height of the stalk, number of internodes, internode diameter and average internode length were recorded and calculated (Table 1). A *z* score was calculated based on the average results of the (ZmUbi-nptII-nos) UKN transgenic control plants and any RNAi plant with a *z* score greater than 2 or −2 (more than two standard deviations from the control group) were considered different to controls. Overall, there were few phenotypic differences detected between RNAi plants and controls. CCoAOMT-5 was the only plant shorter than controls, and this plant also had decreased internode length and smaller internode diameters. COMT-4 had a larger internode diameter than the control group, and COMT-2 had shorter internodes. F5H-4 had more internodes that were shorter than that of the control group.

**Table 1:** Phenotypic measurements of *CCoAOMT*, *F5H* and *COMT* RNAi sugarcane

Plant		Height (cm)		Total number of internodes		Third internode diameter (mm)		Average internode length (cm)	
			*z* score		*z* score		*z* score		*z* score
Control		155.17 ± 28.23		19.67 ± 1.89		12.72 ± 0.73		7.89 ± 1.20	
CCoAOMT	11	163	0.28	21	0.71	12.13	−0.81	7.76	−0.10
	5	80	−*2.66*	20	0.18	11.22	−*2.05*	4.00	−*3.23*
	10	150	−0.18	18	−0.88	12.65	−0.10	8.33	0.37
	9	105	−1.78	17	−1.41	11.90	−1.12	6.18	−1.42
F5H	4	131	−0.86	24	*2.30*	11.79	−1.27	5.46	−*2.02*
	2	130	−0.89	18	−0.88	12.83	0.15	7.22	−0.55
	7	104	−1.81	16	−1.94	12.70	−0.03	6.50	−1.15
	1	151	−0.15	22	1.24	13.01	0.40	6.86	−0.85
COMT	2	107	−1.71	22	1.24	11.69	−1.41	4.86	−*2.51*
	10	113	−1.49	16	−1.94	12.57	−0.20	7.06	−0.68
	3	172	0.60	20	0.18	12.92	0.27	8.60	0.59
	4	139	−0.57	19	−0.35	14.65	*2.63*	7.32	−0.47

Overall averages for controls (*n* = 6 individual plants) are presented with standard deviation. *Z* scores represent the number of standard deviations each RNAi plant measurement is from the control average, with *z* scores greater than 2 or less than −2 highlighted in italic font. Plants are listed in ascending order of total lignin content

### Cell wall composition and structure

**Fig. 2:**
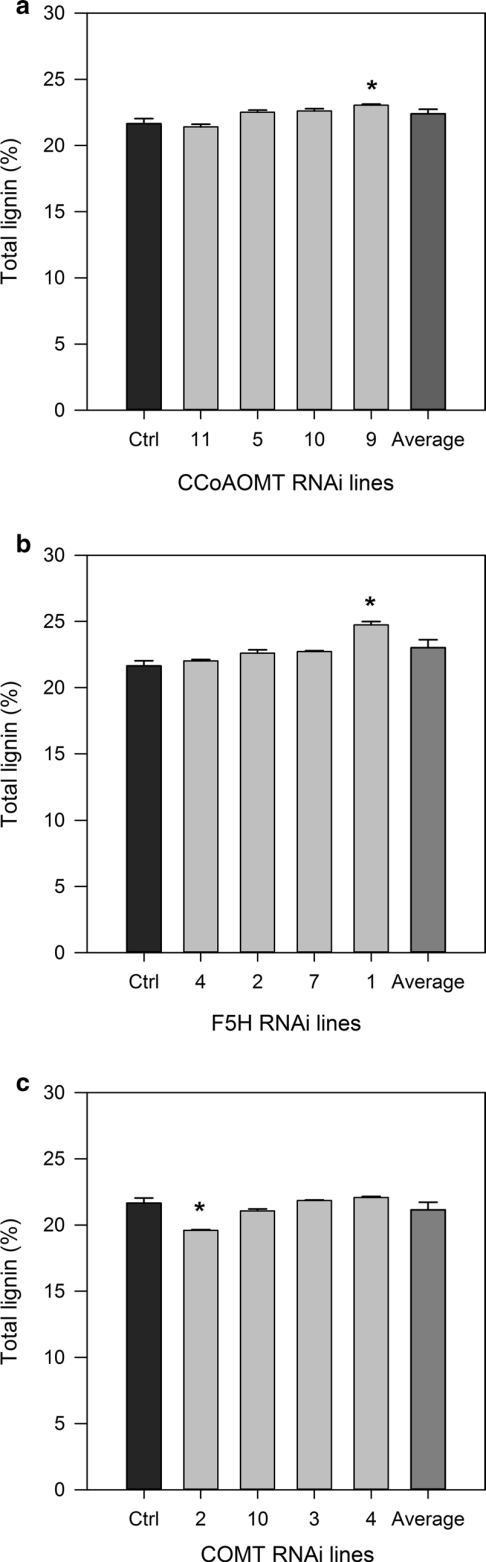
Total lignin content (% dry weight) of RNAi transgenic sugarcane. Total lignin content of individual RNAi sugarcane plants and averages showing standard error of the mean. **a** CCoAOMT, **b** F5H, and **c** COMT. Samples significantly different to the controls, *p* = 0.05, are shown with asterisk. Control *n* = 3. Data on all lines and statistical analysis are displayed in Additional file 3

Four plants per construct which showed the greatest reduction in the expression of the RNAi targeted gene in the maturing internode tissue were selected for cell wall compositional analysis. The majority of the RNAi plants across the three construct lines had total lignin contents similar to that of controls (Fig. 2; Additional file 3). Exceptions include CCoAOMT-9 and F5H-1 that had significantly increased lignin and COMT-2 with significantly reduced lignin (Fig. 2). In all three plants, this corresponded to significant changes in the acid-insoluble lignin content of these plants (Additional file 3).

There were also some changes to structural carbohydrate contents found in RNAi lines (Additional file 3). Two lines, CCoAOMT-10 and F5H-2 had reduced glucose levels. CCoAOMT-9 had reduced arabinose levels and CCoAOMT-5 had reduced xylose content. Three of four F5H lines had reduced arabinose, and F5H-2 also had reduced xylose levels. Only one COMT line, COMT-10, had altered carbohydrate content with a significant reduction in arabinose.

Limited bagasse allowed for only some samples to undergo determination of cellulose crystallinity index. Statistical analysis was not performed as the limited number of samples (one control plant and four RNAi plants) would not provide reliable results. The control had a cellulose crystallinity of 53.5% and the transgenic lines ranged from 53.9 to 57.9% (Additional file 4).

### Enzymatic hydrolysis

**Fig. 3:**
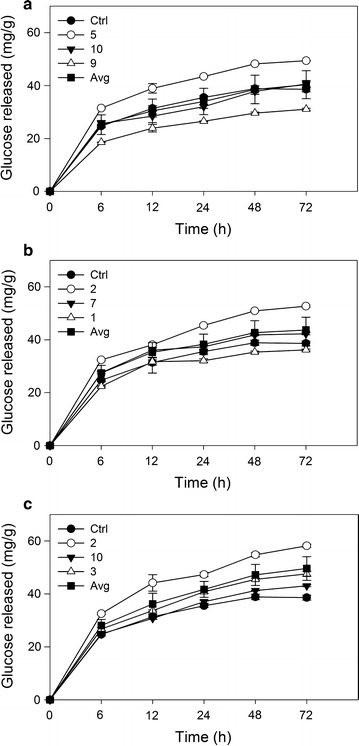
Enzymatic hydrolysis of RNAi transgenic sugarcane. Total glucose concentration in enzymatic hydrolysis solution (mg/mL) per gram (g) of bagasse for individual RNAi plants and averages, showing standard error of the mean measured at six time points over an incubation period of 72 h for **a** CCoAOMT, **b** F5H, and **c** COMT using values from Additional file 5. Control *n* = 3

Three lines per construct, with the greatest variation in lignin content from that of the control plants, underwent limited-extent enzymatic hydrolysis. Each RNAi construct had at least one plant that released significantly more glucose than controls. CCoAOMT-5, F5H2, COMT-2 and COMT-3 all released significantly more glucose and CCoAOMT-9 released significantly less (Fig. 3; Additional file 5). The remaining plants in each line were comparable with control plants. Glucose in the cell wall of pretreated bagasse and glucose released after 72 h enzymatic hydrolysis were not well correlated (*R* ^2^ = 0.384).

All lines (CCoAOMT-5, F5H-2, COMT-2 and COMT-3) that released significantly more glucose after 72 h of enzymatic hydrolysis showed significant levels of glucose being released for earlier time points (Fig. 3; Additional file 5), indicating an increased rate of glucose conversion. Furthermore, after the 48 h time point the glucose released by the controls plateaus, whereas the glucose released by these four RNAi plants continues to increase (Fig. 3). One plant (CCoAOMT-9) released significantly less glucose than the controls at each time point (Additional file 5), thus significantly reducing its rate of glucose conversion.

### Soluble sucrose content of extracted juice

**Fig. 4:**
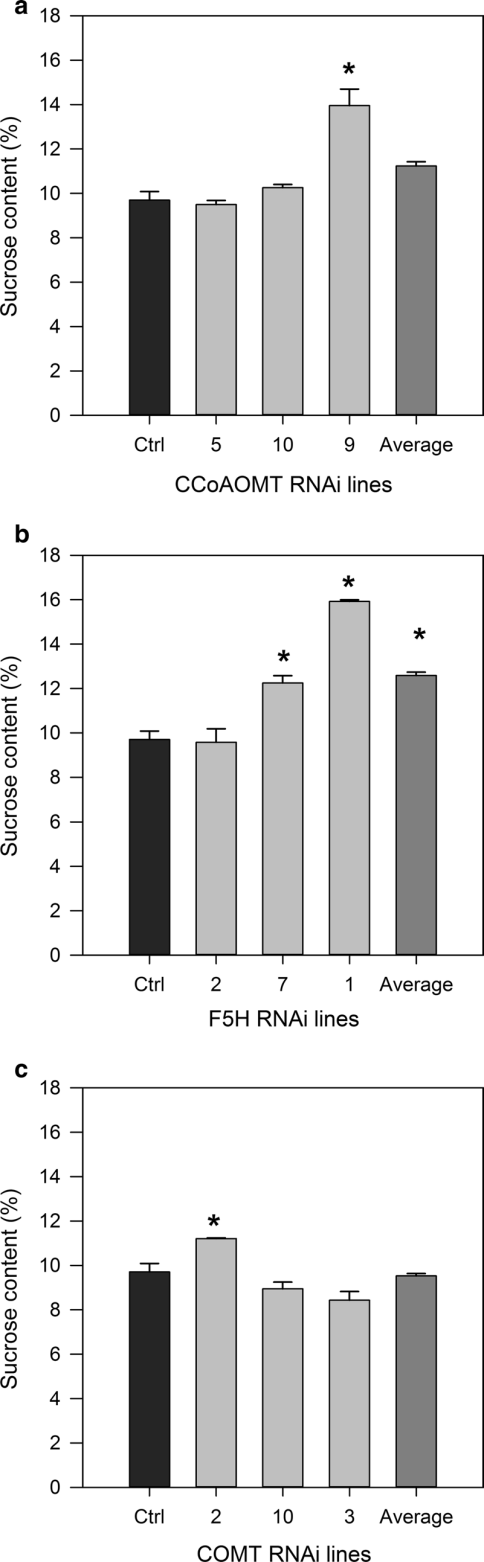
Sucrose content of extracted juice of transgenic sugarcane plants. Sucrose content (% fresh weight) of extracted juice of individual transgenic sugarcane plants and averages selected for enzymatic hydrolysis showing standard error of the mean. **a** CCoAOMT, **b** F5H, and **c** COMT. An *asterisk* indicates a significant difference to controls, *p* = 0.05. Control *n* = 3

The control and RNAi plants were also assessed for sucrose content of extracted juice to determine if the changes in lignin content or structure had affected juice composition and quantity. Two *F5H* RNAi plants (1 and 7) and CCoAOMT-9 showed a significant increase in sucrose levels relative to controls (Fig. 4). COMT-3 had significantly reduced sucrose in extracted juice. All other plants were consistent with controls.

### Lignin structure and composition

**Fig. 5:**
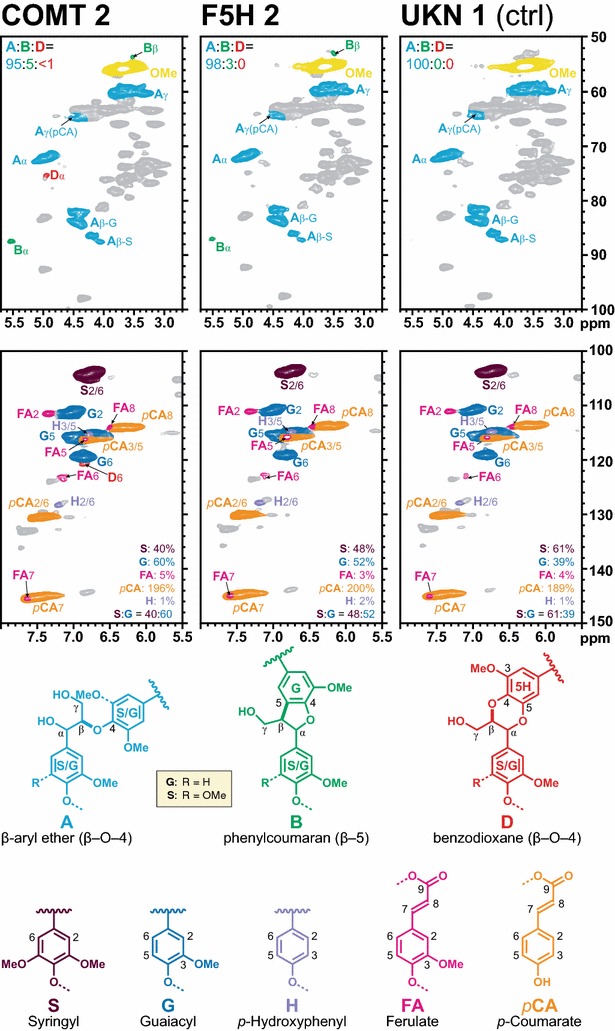
Partial 2D HSQC NMR spectra. HSQC NMR spectra from enzymatic lignins in DMSO-d_6_ from the COMT-2 and F5H-2 transgenic sugarcane bagasse, along with the control. The *top row* shows the aliphatic region, with *color*-*coded* assignments of the main lignin to the *same*-*colored* corresponding structures. The *bottom row* of the aromatic and *double*-*bond region* shows the lignin polymer H, G, and S structural units along with ferulates (mainly from feruloylated arabinoxylan polysaccharides) and *p*-coumarates (on both lignin and polysaccharides); again, contour coloration matches that of the structures shown. The aromatic unit quantification values are from volume integration, on an S + G = 100% basis; accuracy is generally good for the G:S units, but *p*-coumarate and ferulate endgroups are severely overestimated so their integrals should be used only for comparative purposes [74]

Lines with significant changes in glucose release by enzymatic hydrolysis and with enough tissue remaining underwent lignin analysis by NMR. The controls had an S:G of 61:39, whereas the transgenic lines had a decreased S:G, with COMT-2 at 40:60 and F5H-2 at 48:52 (Fig. 5). Phenylcoumaran (β–5-linked) units **B** were found in the COMT-2 and F5H-2 transgenic plants, but not in the controls. Additionally, small amounts of the signature benzodioxane units **D**, derived from the incorporation of 5-hydroxyconiferyl alcohol into the polymer [57–59], were present in the lignins of COMT-2 samples.

## Discussion

Improvement in lignocellulosic biomass quality is essential for cost-competitive bioethanol production [60]. Sugarcane provides a unique advantage over many biomass feedstocks, as it is already transported to a central location for processing [1]. The overall aim of this research was to improve the enzymatic digestibility of bagasse from a commercial sugarcane cultivar by altering lignin deposition and composition. These alterations were achieved by employing RNAi to specifically target and reduce the expression of three lignin biosynthetic genes: *CCoAOMT*, *F5H* and *COMT*. Previous research in other species found that the downregulation of these genes can alter the lignin polymer, as well as reduce the overall deposition of lignin, which has led to improved saccharification and in both dicot and monocot species [4, 31, 32, 61].

Despite reductions in the expression of each of the lignin biosynthetic genes in the respective transgenics, there were few lines with altered cell wall composition. *CCoAOMT* RNAi plants selected for compositional analysis showed reduced levels of *CCoAOMT* by 83–97% in maturing tissue, but no plants were found to have decreased lignin content, and in fact one line, CCoAOMT-9, showed significant increases in lignin content (Additional file 3). In young stem tissue, *CCoAOMT* expression was less reduced than in maturing stem (Additional file 2). None of the *F5H* RNAi plants had decreased lignin content, and again, one line had increased lignin. The *F5H* RNAi sugarcane plants analyzed had minimal to no reductions of *F5H* expression in leaf and young internode tissue yet three of the four plants had reductions of 71–84% in *F5H* expression in the maturing stem (Additional file 2). Despite little change in lignin content in the transgenic lines, there was a significant change in the lignin monomer ratio in favor of the *G* subunit. The reduction of *S* monomers associated with the downregulation of *F5H* and *COMT* may improve enzymatic hydrolysis despite there being no reduction in lignin content, and the introduction of novel 5-hydroxyconiferyl alcohol monomers into the lignin in COMT-deficient plants also needs to be taken into account. This trade-off may allow for improved glucose release without the negative impacts on phenotype that sometimes arise from dramatic reductions in lignin content.

As mentioned, F5H-1 also showed a significant increase in lignin content, similar to previous work showing an increase in lignin content in an *f5* *h* mutant Arabidopsis [21]. The remaining *F5H* RNAi plants had no changes in lignin content, similar to results in alfalfa [12, 13, 23, 24] and Arabidopsis [21] where reduced F5H expression did not lead to decreased lignin content. Lignin level changes are less anticipated for genes late in the pathway that change the distribution of lignin monomer units but not necessarily the amount of polymer synthesis.

There was variation in the expression levels of *COMT* across the *COMT* RNAi events (Fig. 1; Additional file 2). COMT-2 had a 59% decrease in *COMT* expression in leaf tissue, little change in young internode tissue and a fourfold increase in *COMT* expression in maturing tissue. COMT-10 and COMT-4 had higher expression levels in young internode and reduced expression levels in maturing tissue. Previous work reported significant decreases in *COMT* expression in internode three of *COMT* RNAi sugarcane [31, 32]. Although decreases in *COMT* expression were found in maturing internodes of COMT-10 and -4 in this study, these decreases were not significant, and are minimal relative to published results [31, 32]. The same *COMT* accession was used for construct design (AJ231133) and there was approximately 250 bp overlap between the RNAi target sequence used in this study and in the published studies [31, 32]. The sequence targeted in the previous studies is further upstream than the sequence targeted in this research, resulting in different sequences of the SAM-binding pockets being targeted by the non-overlapping regions, which may have increased the effectiveness of the RNAi construct [31, 32]. The differences in COMT downregulation may be attributed to different promoters, with previous work using the *Os*C4H promoter, *Pn*4CL spacer intron and the CaMV 35S terminator as opposed to the *Zm*Ubi promoter, syntron spacer intron and *nos* terminator used in this current research.

COMT-2 was the only plant across the three RNAi lines with a significant reduction in total lignin content (Fig. 2). Although COMT-10 and COMT-4 had reduced *COMT* expression in maturing stem, they had expression levels higher than controls in young tissue, which as previously discussed, may have allowed lignin polymer synthesis and deposition to occur before the reduction of *COMT* expression. The 9.5% decrease in lignin content in COMT-2 is within the range of lignin reductions previously reported by RNAi targeting of *COMT*. Jung et al. found lignin reductions of 3.9–13.7% in greenhouse-grown sugarcane [31] and 5.5–12% reductions in field-grown sugarcane [32]. RNAi targeting of *COMT* reduced lignin content by 6.4–14.7% in switchgrass [4, 29, 30]. Other research has reported greater reductions of lignin content of 20% in alfalfa [13], 35% in *Brassica napus* [62] and 40% in canola [28].

There were few lines with improved glucose release by enzymatic hydrolysis (Additional file 5). The one COMT line (COMT-2) with reduced lignin had significantly increased glucose release, and one F5H line (F5H-2) had increased glucose release. Interestingly, this F5H line also had significantly decreased structural glucose levels. Both of these lines with improved glucose release had significantly decreased *S:G* ratios (Fig. 5), supporting the role of lignin monomer ratio having an effect on cellulose accessibility. More importantly, in this COMT-deficient line is that *S:G* ratios only tell part of the story—it completely ignores the important fact that these lignins are structurally dramatically altered by the incorporation of the novel 5-hydroxyconiferyl alcohol monomer into the lignins, creating novel benzodioxane structures in the lignin [9, 57, 59, 63–68]. Such benzodioxane units *D* were detected in the lignin from COMT-2 sample, but only at low levels. Such structures are also present in the seed coats and one plant in particular, *Escobaria dasyacantha*, has its seed coat lignin derived entirely from 5-hydroxyconiferyl alcohol [69]. The presence of benzodioxane unit indicates that the suppression of COMT here successfully reduces the methylation reaction.

Previous research found reduced expression of *CCoAOMT* improves saccharification in Arabidopsis [21] and alfalfa [12]. Similarly, although no differences in lignin content were detected in CCoAOMT-5, this plant released 28% more glucose than the controls after 72 h. CCoAOMT may also be involved in the production of ferulate residues that aid in cross-linking cell wall components contributing to structural stability, and therefore the recalcitrant nature of cell walls to enzymatic hydrolysis [11, 70]. A reduction in CCoAOMT activity may in turn reduce the production of ferulates, resulting in reduced cross-linkages in the cell wall, increased susceptibility of the cell wall to enzymatic degradation, and improved saccharification [3, 70, 71].

COMT-2 and COMT-3 released significantly more glucose after 72 h, consistent with the results in alfalfa [12], switchgrass [4, 29] and sugarcane [31, 32]. COMT-2 was the only plant with a significant reduction in total lignin content, which may partially explain the 51% increase in glucose release. The improved glucose release by COMT-3, which had a lignin content equivalent to that of the controls, may again be explained by the incorporation of 5-hydroxyconiferyl alcohol into the polymer, an hypothesis proposed [4, 7, 33, 34, 63, 69, 72], although this has not been confirmed experimentally.

For lignin-reduced sugarcane to remain commercially viable it is important that the reduction in lignin level or the alterations to cell wall composition do not produce detrimental phenotypes, nor affect the juice sucrose content of the sugarcane as the carbon flux directed for sucrose synthesis may be affected by the partitioning of carbon for cell wall synthesis [73]. The plants that underwent enzymatic hydrolysis were also assessed for juice sugar content. Two of the *F5H* RNAi plants, F5H-7 and F5H-1, along with CCoAOMT-9 had significant increases in sucrose levels (Fig. 4). Only one line, COMT-3, had reduced levels of sucrose. Jung et al. found that two COMT RNAi sugarcane plants had soluble solids (Brix) levels comparable with controls and two plants had significant reductions. Brix is a measurement of soluble solids in extracted juice and an estimate of sucrose levels [32, 73]. Lines with significantly increased sucrose levels are of great commercial interest as this would add significant monetary value to these sugarcane plants, even before the use of bagasse for lignocellulosic fuels. *COMT* and *PAL* were up-regulated in high brix plants, and additionally, PAL expression was shown to be inducible by sucrose [73]. The authors suggested that increased sucrose may induce lignin biosynthesis which could explain the significant increase in lignin in F5H-1 [73], and that this link may provide direction for future research if manipulations to lignin biosynthesis can also influence sucrose content.

## Conclusions

This research employed RNAi to specifically downregulate the expression of *CCoAOMT*, *F5H* and *COMT*, three genes within the lignin biosynthetic pathway, with the aim of altering lignin deposition and composition and improving the release of glucose after enzymatic hydrolysis for second-generation bioethanol production. One plant from each of the *CCoAOMT* and *F5H* RNAi lines and two plants from the *COMT* RNAi line released significantly more glucose after enzymatic hydrolysis. Of the four plants, only COMT-2 had a significant reduction in lignin, along with altered lignin monomer composition. In some lines, sucrose levels actually increased, providing evidence that the modification of lignin biosynthesis to improve the quality of bagasse for biofuels may be a complementary condition to the enhanced production of sugar and other current commodities.

## Additional files

**Additional file 1: Table S1.** Primers for cloning, screening and qRT-PCR. Primers 1-3 were used for PCR fragment amplification of sugarcane *CCoAOMT*, *F5H* and *COMT* gene sequences for RNAi vector construction. Primers introduce a *SmaI* restriction site at 5′ end of PCR fragment (*underlined*). Primers 4–6 were designed for screening of CCoAOMT, F5H and COMT RNAi regenerated events and qRT-PCR quantification of targeted gene expression levels.

**Additional file 2: Table S2.** Normalized qRT-PCR ΔCt values of gene expression levels of RNAi targeted lignin biosynthetic genes. Values represent initial screening of leaf tissue and post-harvest expression results from young internode tissue and maturing internode tissue. All data normalized against transgenic controls with standard error of the mean shown. Samples significantly different to controls after a one-way ANOVA, *p* < 0.05 are shown in bold. NE: Normalized expression.

**Additional file 3: Table S3.** Cell wall composition of CCoAOMT, F5H and COMT RNAi sugarcane plants. The percentage of each component of the total composition is shown with the standard error of the mean. Samples significantly different to the transgenic controls after one-way ANOVA, *p* < 0.05, are shown in bold. Plants are listed in ascending order of total lignin content. Control *n* = 6. Avg is the mean of the lines within each construct.

**Additional file 4: Table S4.** Cellulose crystallinity index of RNAi bagasse. Crystallinity index was calculated using the height ratio between the intensity of the crystalline peak (I_002_ − I_AM_) and the total intensity (I_002_) following subtraction of the background signal.

**Additional file 5: Table S5.** Glucose (mg/g) released via limited enzymatic hydrolysis. Glucose released per gram of bagasse measured at six time points for *CCoAOMT*, *F5H* and *COMT* RNAi plants. The glucose released is shown with the standard error of the mean. Samples significantly different to controls after a one-way ANOVA, *p* < 0.05 are shown in bold. Plants for each line are listed in ascending order of total lignin content. Avg is the mean of the lines within each construct.

## Abbreviations

4CL: 4-coumarate CoA ligase
ANOVA: analysis of variance
C4H: cinnamate 4-hydroxylase
CAD: cinnamyl alcohol dehydrogenase
CaMV: cauliflower mosaic virus
CCoAOMT: caffeoyl-CoA *O*-methyltransferase
CCR: cinnamoyl-CoA reductase
COMT: caffeic acid *O*-methyltransferase
DMSO: dimethyl sulfoxide
EST: expressed sequence tags
F5H: ferulate 5-hydroxylase
FPU: filter paper unit
G: guaiacyl lignin
HSQC: heteronuclear single-quantum coherence spectroscopy
HPIC: high performance ion chromatography
HPLC: high performance liquid chromatography
LSD: least significant difference
NMR: nuclear magnetic resonance
NOS: nopaline synthase
PAL: phenylalanine ammonia-lyase
qRT-PCR: quantitative PCR
RNAi: RNA interference
S: syringyl lignin
SAM: *S*-adenosyl-l-methionine
UBI: ubiquitin
UKN: ZmUbi-nptII-nos
