# Supplementary material for: RNAi downregulation of three key lignin genes in sugarcane improves glucose release without reduction in sugar production
Source: Biotechnol Biofuels. 2016 Dec 20;9:270. doi: 10.1186/s13068-016-0683-y (PMC5168864; doi:10.1186/s13068-016-0683-y)
Supplement: Supplementary file 1 — Additional file 1: Table S1. Primers for cloning, screening and qRT-PCR. Primers 1-3 were used for PCR fragment amplification of sugarcane CCoAOMT, F5H and COMT gene sequences for RNAi vector construction. Primers introduce a SmaI restriction site at 5′ end of PCR fragment (underlined). Primers 4–6 were designed for screening of CCoAOMT, F5H and COMT RNAi regenerated events and qRT-PCR quantification of targeted gene expression levels. [file 13068_2016_683_MOESM1_ESM.docx]

**TABLE S1:** **Primers for cloning, screening and qRT-PCR.** Primers 1-3 were used for PCR fragment amplification of sugarcane *CCoAOMT*, *F5H* and *COMT* gene sequences for RNAi vector construction. Primers introduce a *SmaI* restriction site at 5' end of PCR fragment (underlined). Primers 4-6 were designed for screening of CCoAOMT, F5H and COMT RNAi regenerated events and qRT-PCR quantification of targeted gene expression levels.

| **No** | **Amplicon** | **Forward (5'-3')** | **Reverse (5'-3')** | **Size (bp)** |
| --- | --- | --- | --- | --- |
| 1 | CCoAOMT | CCCGGGGACCTCTACCAGTACATCCTGGAC | CGTCCACGAAGACGAAGTCGAAC | 415 |
| 2 | F5H | CCCGGGCTCAAGTGCGTCATCAAGGAGAC | AAGATGTCGCCCATGTCCAGCTC | 401 |
| 3 | COMT | CCCGGGCACGGACCCGCGCTTCAACCGC | CAGCACGCACTCGACGACGATC | 406 |
| 4 | CCoAOMT | ACCTCATCGCAGACGAGAAGAAC | AGCCGCTCGTGGTAGTTGAGGTAG | 91 |
| 5 | F5H | GGTTCATCGACAAGATCATCGAC | GTCGGGGCTCTTCCCGCGCTTCAC | 53 |
| 6 | COMT | TACGGGATGACGGCGTTCGAGTAC | GTGATGATGACCGAGTGGTTCTT | 92 |
