# Supplementary material for: RNAi downregulation of three key lignin genes in sugarcane improves glucose release without reduction in sugar production
Source: Biotechnol Biofuels. 2016 Dec 20;9:270. doi: 10.1186/s13068-016-0683-y (PMC5168864; doi:10.1186/s13068-016-0683-y)
Supplement: Supplementary file 2 — Additional file 2: Table S2. Normalized qRT-PCR ΔCt values of gene expression levels of RNAi targeted lignin biosynthetic genes. Values represent initial screening of leaf tissue and post-harvest expression results from young internode tissue and maturing internode tissue. All data normalized against transgenic controls with standard error of the mean shown. Samples significantly different to controls after a one-way ANOVA, p < 0.05 are shown in bold. NE: Normalized expression. [file 13068_2016_683_MOESM2_ESM.docx]

TABLE S2: Normalized qRT-PCR ΔCt values of gene expression levels of RNAi targeted lignin biosynthetic genes. Values represent initial screening of leaf tissue and post-harvest expression results from young internode tissue and maturing internode tissue. All data normalized against transgenic controls with standard error of the mean shown. Samples significantly different to controls after a one-way ANOVA, *p* <0.05 are shown in bold. NE: Normalized expression.

|  |  | **Leaf** | | **Young** | | **Maturing** | |
| --- | --- | --- | --- | --- | --- | --- | --- |
| Plant | Event | NE | +/- | NE | +/- | NE | +/- |
| Control |  | 1.00 | 0.10 | 1.00 | 0.14 | 1.00 | 0.37 |
| CCoAOMT | 9 | 1.03 | 0.01 | 0.98 | 0.02 | **0.03** | **0.00** |
|  | 10 | **0.31** | 0.00 | 0.98 | 0.01 | **0.09** | **0.00** |
|  | 5 | **0.20** | 0.00 | **0.54** | **0.01** | **0.14** | **0.00** |
|  | 11 | 0.95 | 0.02 | 1.00 | 0.01 | **0.17** | **0.00** |
|  | 1 | **1.44** | **0.17** | 1.05 | 0.02 | **0.30** | **0.01** |
|  | 13 | **1.69** | **0.06** | 1.07 | 0.03 | 0.39 | 0.00 |
|  | 7 | **0.69** | **0.01** | 1.02 | 0.03 | 1.53 | 0.10 |
|  | 2 | **0.63** | **0.01** | **0.55** | **0.02** | **1.76** | **0.03** |
|  | 8 | **0.56** | **0.02** | **1.73** | **0.04** | **1.89** | **0.03** |
| F5H | 7 | 0.76 | 0.05 | **5.40** | **0.81** | **0.16** | **0.00** |
|  | 2 | 1.23 | 0.20 | 0.83 | 0.02 | **0.17** | **0.00** |
|  | 4 | 0.74 | 0.03 | 0.85 | 0.01 | 0.29 | 0.04 |
|  | 1 | 1.06 | 0.08 | 0.84 | 0.01 | 0.96 | 0.01 |
|  | 13 | 0.79 | 0.12 | 0.74 | 0.04 | 1.12 | 0.06 |
|  | 3 | 0.72 | 0.03 | 0.77 | 0.04 | 1.23 | 0.02 |
|  | 14 | **1.29** | **0.05** | **1.33** | **0.02** | **1.98** | **0.04** |
|  | 6 | 0.93 | 0.14 | **1.35** | **0.02** | **2.18** | **0.02** |
|  | 8 | **0.57** | **0.09** | **1.77** | **0.07** | **4.75** | **0.05** |
| COMT | 4 | **1.37** | **0.02** | **3.82** | **0.12** | 0.68 | 0.01 |
|  | 10 | **2.33** | **0.13** | **1.80** | **0.03** | 0.79 | 0.03 |
|  | 3 | 1.19 | 0.05 | 0.88 | 0.01 | 1.26 | 0.02 |
|  | 2 | **0.41** | **0.06** | 0.98 | 0.02 | **4.01** | **0.11** |
|  | 13 | **3.00** | **0.07** | **14.06** | **0.48** | **4.23** | **0.12** |
|  | 8 | **2.09** | **0.12** | **2.25** | **0.03** | **5.27** | **0.17** |
|  | 14 | **2.92** | **0.06** | **7.72** | **0.17** | **6.59** | **0.11** |
|  | 7 | **1.61** | **0.06** | **4.40** | **0.08** | **10.90** | **0.07** |
