# Supplementary material for: RNAi downregulation of three key lignin genes in sugarcane improves glucose release without reduction in sugar production
Source: Biotechnol Biofuels. 2016 Dec 20;9:270. doi: 10.1186/s13068-016-0683-y (PMC5168864; doi:10.1186/s13068-016-0683-y)
Supplement: Supplementary file 3 — Additional file 3: Table S3. Cell wall composition of CCoAOMT, F5H and COMT RNAi sugarcane plants. The percentage of each component of the total composition is shown with the standard error of the mean. Samples significantly different to the transgenic controls after one-way ANOVA, p < 0.05, are shown in bold. Plants are listed in ascending order of total lignin content. Control n = 6. Avg is the mean of the lines within each construct. [file 13068_2016_683_MOESM3_ESM.docx]

**TABLE 3:** **Cell wall composition of CCoAOMT, F5H and COMT RNAi sugarcane plants.** The percentage of each component of the total composition is shown with the standard error of the mean. Samples significantly different to the transgenic controls after one-way ANOVA, *p* < 0.05, are shown in bold. Plants are listed in ascending order of total lignin content. Control n = 6. Avg is the mean of the lines within each construct.

|  |  | Total lignin | | Acid insoluble | | Acid soluble | | Glucose | | Xylose | | Galactose | | Arabinose | |
| --- | --- | --- | --- | --- | --- | --- | --- | --- | --- | --- | --- | --- | --- | --- | --- |
|  |  |  |  | lignin | | lignin | |  |  |  |  |  |  |  |  |
|  |  | % | +/- | % | +/- | % | +/- | % | +/- | % | +/- | % | +/- | % | +/- |
| Control |  | 21.65 | 0.38 | 17.13 | 0.38 | 4.53 | 0.09 | 49.83 | 0.55 | 23.53 | 0.25 | 0 | 0 | 2.03 | 0.08 |
| CCoAOMT RNAi | 11 | 21.4 | 0.2 | 16.83 | 0.14 | 4.57 | 0.07 | 52.04 | 0.91 | 23.43 | 0.5 | 0 | 0 | 1.92 | 0.04 |
|  | 5 | 22.51 | 0.16 | 17.97 | 0.2 | 4.54 | 0.06 | 50.04 | 1.01 | **20.37** | **0.42** | 0 | 0 | 1.65 | 0.13 |
|  | 10 | 22.61 | 0.17 | 18.07 | 0.13 | 4.55 | 0.08 | **47.9** | **0.35** | 23.37 | 0.17 | 0 | 0 | 2.07 | 0.01 |
|  | 9 | **23.05** | **0.08** | **18.48** | **0.02** | 4.58 | 0.09 | 49.14 | 0.78 | 22.76 | 0.44 | 0 | 0 | **1.68** | **0.06** |
|  | Avg | 22.39 | 0.35 | 17.84 | 0.35 | 4.56 | 0.01 | 49.78 | 0.87 | 22.48 | 0.72 | 0 | 0 | 1.83 | 0.1 |
| F5H RNAi | 4 | 22.02 | 0.1 | 17.56 | 0.07 | 4.45 | 0.11 | 49.38 | 0.96 | 21.81 | 0.49 | 0 | 0 | **1.6** | **0.01** |
|  | 2 | 22.61 | 0.25 | 17.97 | 0.19 | 4.65 | 0.07 | **47.31** | **0.27** | **22.37** | **0.16** | 0 | 0 | **1.73** | **0.03** |
|  | 7 | 22.72 | 0.07 | 18.07 | 0.08 | 4.65 | 0.02 | 48.92 | 1.14 | 21.96 | 0.45 | 0 | 0 | 1.76 | 0.07 |
|  | 1 | **24.74** | **0.25** | **20.58** | **0.16** | 4.16 | 0.09 | 49.55 | 0.59 | 23 | 0.28 | 0 | 0 | **1.54** | **0.02** |
|  | Avg | 23.02 | 0.59 | 18.55 | 0.69 | 4.48 | 0.12 | 48.79 | 0.51 | 22.29 | 0.27 | 0 | 0 | 1.66 | 0.05 |
| COMT RNAi | 2 | **19.59** | **0.06** | **15.01** | **0.06** | 4.58 | 0.09 | 51.07 | 0.92 | 23.1 | 0.45 | 0 | 0 | 1.87 | 0.04 |
|  | 10 | 21.06 | 0.15 | 16.62 | 0.02 | 4.45 | 0.14 | 51.56 | 1.19 | 23.04 | 0.51 | 0 | 0 | **1.63** | **0.02** |
|  | 3 | 21.85 | 0.04 | 17.13 | 0.17 | 4.72 | 0.14 | 49.64 | 0.99 | 24.1 | 0.46 | 0 | 0 | 1.82 | 0.01 |
|  | 4 | 22.08 | 0.08 | 17.41 | 0.06 | 4.67 | 0.06 | 48.82 | 0.2 | 23.31 | 0.18 | 0 | 0 | 1.96 | 0.09 |
|  | Avg | 21.15 | 0.56 | 16.54 | 0.54 | 4.61 | 0.06 | 50.27 | 0.63 | 23.39 | 0.24 | 0 | 0 | 1.82 | 0.07 |
