# Supplementary material for: RNAi downregulation of three key lignin genes in sugarcane improves glucose release without reduction in sugar production
Source: Biotechnol Biofuels. 2016 Dec 20;9:270. doi: 10.1186/s13068-016-0683-y (PMC5168864; doi:10.1186/s13068-016-0683-y)
Supplement: Supplementary file 4 — Additional file 4: Table S4. Cellulose crystallinity index of RNAi bagasse. Crystallinity index was calculated using the height ratio between the intensity of the crystalline peak (I002 − IAM) and the total intensity (I002) following subtraction of the background signal. [file 13068_2016_683_MOESM4_ESM.docx]

**TABLE S4: Cellulose crystallinity index of RNAi bagasse**. Crystallinity index was calculated using the height ratio between the intensity of the crystalline peak (I_002_ - I_AM_) and the total intensity (I_002_) following subtraction of the background signal.

| Plant | Event | Crystallinity Index (%) |
| --- | --- | --- |
| Control | 4 | 53.5 |
| CCoAOMT | 9 | 53.9 |
|  | 10 | 57.9 |
| F5H | 2 | 55.6 |
| COMT | 10 | 54.7 |
