# Supplementary material for: RNAi downregulation of three key lignin genes in sugarcane improves glucose release without reduction in sugar production
Source: Biotechnol Biofuels. 2016 Dec 20;9:270. doi: 10.1186/s13068-016-0683-y (PMC5168864; doi:10.1186/s13068-016-0683-y)
Supplement: Supplementary file 5 — Additional file 5: Table S5. Glucose (mg/g) released via limited enzymatic hydrolysis. Glucose released per gram of bagasse measured at six time points for CCoAOMT, F5H and COMT RNAi plants. The glucose released is shown with the standard error of the mean. Samples significantly different to controls after a one-way ANOVA, p < 0.05 are shown in bold. Plants for each line are listed in ascending order of total lignin content. Avg is the mean of the lines within each construct. [file 13068_2016_683_MOESM5_ESM.docx]

**TABLE S5:** **Glucose (mg/g) released via limited enzymatic hydrolysis.** Glucose released per gram of bagasse measured at six time points for *CCoAOMT*, *F5H* and *COMT* RNAi plants. The glucose released is shown with the standard error of the mean. Samples significantly different to controls after a one-way ANOVA, *p* <0.05 are shown in bold. Plants for each line are listed in ascending order of total lignin content. Avg is the mean of the lines within each construct.

|  |  | 0 hrs | | 6 hrs | | 12 hrs | | 24 hrs | | 48 hrs | | 72 hrs | |
| --- | --- | --- | --- | --- | --- | --- | --- | --- | --- | --- | --- | --- | --- |
|  |  | mg/mL | +/- | mg/mL | +/- | mg/mL | +/- | mg/mL | +/- | mg/mL | +/- | mg/mL | +/- |
| Control |  | 0.0 | 0.0 | 24.69 | 0.56 | 31.38 | 1.03 | 35.50 | 0.68 | 38.86 | 1.04 | 38.58 | 0.96 |
| CCoAOMT-RNAi | 5 | 0.0 | 0.0 | **31.46** | **0.19** | 38.94 | 1.79 | **43.42** | **0.18** | **48.21** | **0.26** | **49.39** | **0.13** |
|  | 10 | 0.0 | 0.0 | 25.67 | 1.18 | 28.45 | 0.32 | 32.01 | 0.63 | 37.82 | 1.13 | 40.59 | 1.34 |
|  | 9 | 0.0 | 0.0 | **18.57** | **0.05** | **23.90** | 1.42 | **26.51** | **0.16** | **29.60** | **0.36** | **31.06** | **0.39** |
|  | Avg | 0.0 | 0.0 | 25.23 | 3.73 | 30.43 | 4.54 | 33.98 | 4.98 | 38.54 | 5.38 | 40.35 | 5.29 |
| F5H-RNAi | 2 | 0.0 | 0.0 | **32.38** | **0.29** | **38.06** | 0.35 | **45.39** | **0.51** | **50.92** | **0.13** | **52.68** | **0.40** |
|  | 7 | 0.0 | 0.0 | **27.53** | **0.36** | 36.06 | 2.89 | 37.30 | 0.85 | 41.83 | 0.18 | 42.27 | 0.38 |
|  | 1 | 0.0 | 0.0 | 22.36 | 0.24 | 31.68 | 4.29 | 32.07 | 0.67 | 35.32 | 0.37 | 36.14 | 0.34 |
|  | Avg | 0.0 | 0.0 | 27.43 | 2.89 | 35.26 | 1.88 | 38.25 | 3.88 | 42.69 | 4.52 | 43.70 | 4.83 |
| COMT-RNAi | 2 | 0.0 | 0.0 | **32.52** | **0.33** | 44.16 | 3.11 | **47.31** | **0.85** | **54.79** | **0.79** | **58.16** | **0.59** |
|  | 10 | 0.0 | 0.0 | 24.87 | 0.23 | 30.75 | 0.30 | 37.04 | 0.56 | 41.27 | 0.05 | 43.05 | 0.32 |
|  | 3 | 0.0 | 0.0 | 26.75 | 0.58 | 33.69 | 0.28 | **40.80** | **0.62** | **45.50** | **0.37** | **47.57** | **0.94** |
|  | Avg | 0.0 | 0.0 | 28.05 | 2.30 | 36.20 | 4.07 | 41.72 | 3.00 | 47.19 | 4.00 | 49.59 | 4.48 |
